# Supplementary material for: Probiotic consumption influences universal adaptive mutations in indigenous human and mouse gut microbiota
Source: Commun Biol. 2021 Oct 18;4:1198. doi: 10.1038/s42003-021-02724-8 (PMC8523657; doi:10.1038/s42003-021-02724-8)
Supplement: Supplementary file 2 — Supplementary information [file 42003_2021_2724_MOESM2_ESM.pdf]

Supplementary information

Probiotic consumption influences universal adaptive mutations in indigenous human and mouse gut microbiota

Chenchen Ma[1#], Chengcheng Zhang[2#], Denghui Chen[3#], Shuaiming Jiang[1], Siyuan Shen[1], Dongxue Huo[1], Shi Huang[4*], Qixiao Zhai[2*], Jiachao Zhang[1*]

1. College of Food Science and Engineering, Key Laboratory of Food Nutrition and Functional Food of Hainan Province, Hainan University, Haikou, 570228, China
2. State Key Laboratory of Food Science and Technology, School of Food Science and Technology, Jiangnan University, Wuxi, 214122,China
3. Department of Psychiatry, University of California San Diego, La Jolla, CA 92093, USA
4. Department of Pediatrics and Center for Microbiome Innovation at Jacobs School of Engineering, University of California San Diego, 9500 Gilman Drive, La Jolla, CA 92093, USA

# These authors contributed equally.

* Correspondence: Jiachao Zhang (E-mail: zhjch321123@163.com), Qixiao Zhai (E-mail: zhaiqixiao@sina.com) and Shi Huang (E-mail: shihuang047@gmail.com)

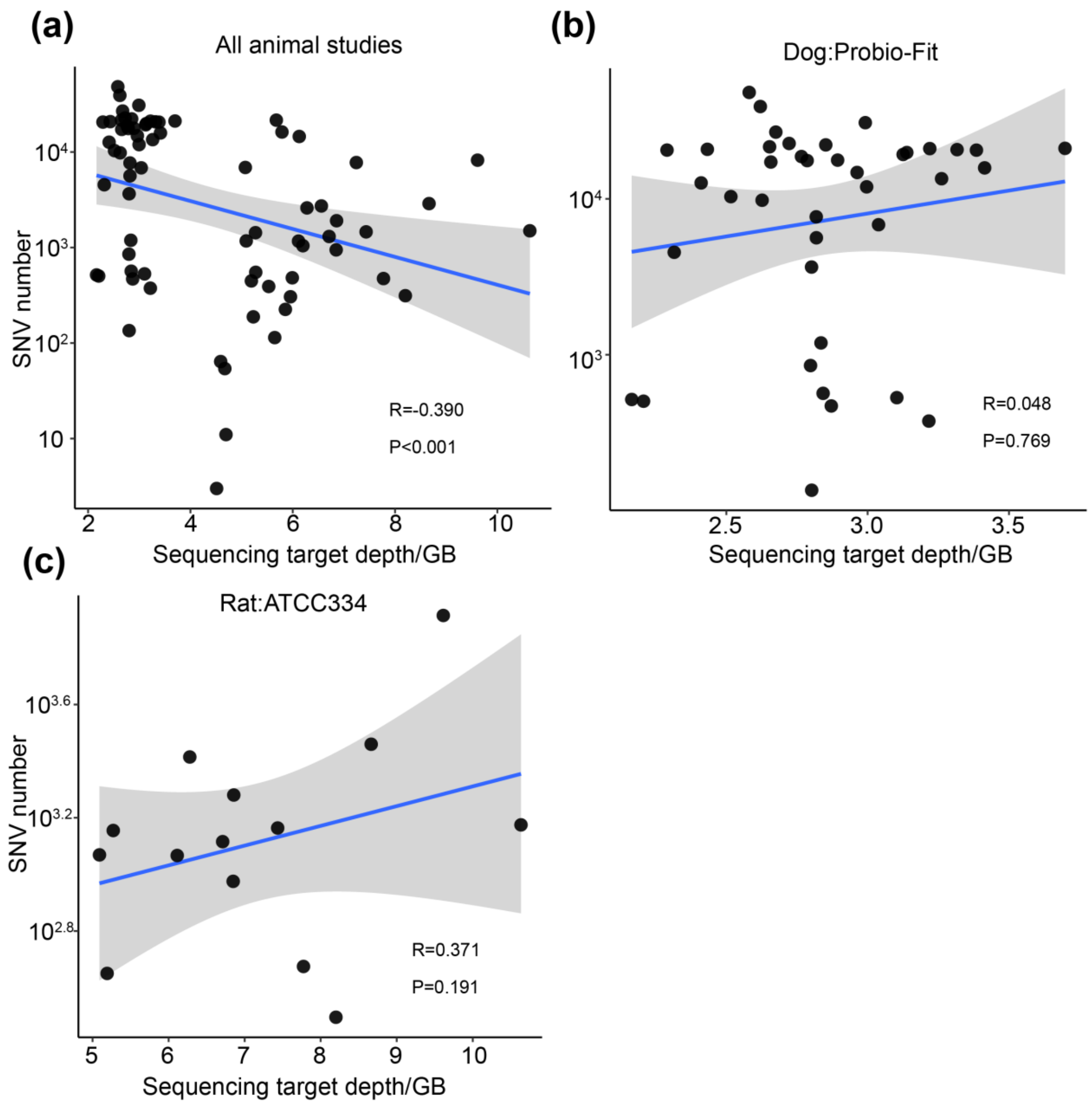

Supplementary figure 1：The associations between SNV number and sequencing depths in different animal models. The x-coordinate is sequencing depths, and the y-coordinate is SNV number. (**a**) The correlation between SNV number and sequencing depths in all animal studies (R=-0.390, p<0.001). (**b**) Only a study (dog) was considered (R=0.048, p=0.769). (**c**) Only a study (rat) was considered (R=0.371, p=0.191).

(a)

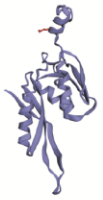

>REF-30S ribosomal protein S5
MAMRNREDDGMITKVVSINRVSKTVKGGRIMKFAALV
VVGDGKGTIGYGIGKSGEVPEAIRKGEAAAKKNMHK
VALKGTTIPHEIVGKYGAGAVLLKPAAPGTGMIAGGP
VRAVIEAAGIKDVRAKSMRSNNPINVVAATFAGLCGLV
SAESVAEKRGKTVKEILG

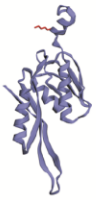

>ALT-30S ribosomal protein S5
MAMRNREDDGMITKVVSINRVSKTVKGGRIMKFAALV
VVGDGKGTIGYGIGKSGEVPEAIRKGEAAAKKNMHKV
ALKGTTIPHEIVGKYGAGAVLLKPAAPGTGMIAGGPVR
AVIEAAGIKDVRAKSMRSNNPINVVAATFAGLCGLVSA
KSVAEKRGKTVKEILG

(b)

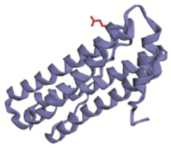

>REF-Ferritin
MMNANVSKLLNEQINKEFYSAYLYLDFANYYAAVGLDGFE
NWYRVQAQEERDHAMLFYQYLQNNGEGVTFEAIAKPEW
ERVDHMTPLKKALEHEKLVTASIDAIYAAAHEVRDFRTMQ
TLDWFIKEQGEEEKNAADLITKMELFGGDSKGLYMLNSEL

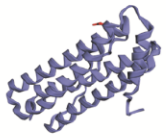

>ALT-Ferritin
MMNANVSKLLNEQINKEFYSAYLYLDFANYYAAVGLDGFE
NWYRVQAQEERDHAMLFYQYLQNNGEGVTFEAIAKPEW
ERVDHMTPLKKALEHEKLVTASIDAIYAAAHEVRDFRTMQT
LDWFIKEQGEEEKNAADLITKMDLFGGDSKGLYMLNSELK

(c)

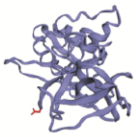

>REF-Peptidase S24
MKKKTTLTKIHIAPSTVRYDAVAARDSNEVGQILAGTRKRNGYSLVA
FSELLRHYGVDVSDKGISKWEKGYTTPSIYQLVAICYALNIKEGPSY
FTKNFQKPALLNDIGQKKVAEYEMDLIASRRYQPDAEEPAEIDYIMM
PVSELPVSAGLGAFLEGEMFQQIQVPASSVPAGAEFGIYVSGDSM
EPRYHNGQIVWVKRCEELKCGDIGIFVYDDCGYLKKYDEHTPDKS
QAEFLTDSYGVVHNQPVLVSLNTKYSPILISPEQRFEVVGKVLN

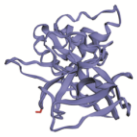

>ALT-Peptidase S24
MKKKTTLTKIHIAPSTVRYDAVAARDSNEVGQILAGTRKRNGYSLV
AFSELLRHYGVDVSDKGISKWEKGYTTPSIYQLVAICYALNIKEGPS
YFTKNFQKPALLNDIGQKKVAEYEMDLIASRRYQPDAEEPAEIDYIM
MPVSELPVSAGLGAFLEGEMFQQIQVPASSVPAGAEFGIYVSGDS
MEPRYHSGQIVWVKRCEELKCGDIGIFVYDDCGYLKKYDEHTPDK
SQAEFLTDSYGVVHNQPVLVSLNTKYSPILISPEQRFEVVGKVLN

Supplementary figure 2: The predicted structures and amino acid sequences of all proteins related in non-synonymous SNVs. (**a**). Gene product: 30S ribosomal protein S5. (**b**) Gene product: Ferritin. (**c**) Gene product: Peptidase S24. The protein structure was (Type II toxin-antitoxin system PemK/MazF family toxin) not predicted successfully.

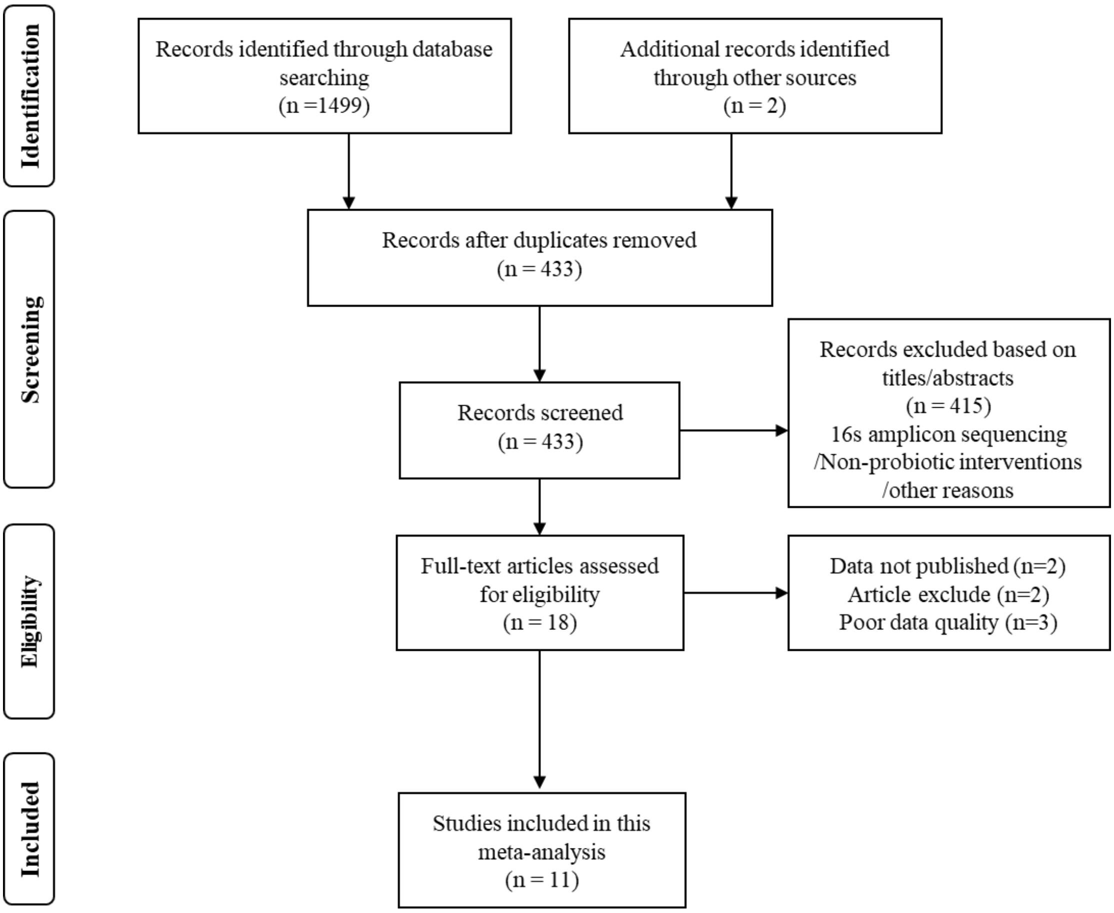

Supplementary figure 3: Flow chart of literature screening and data curation process.

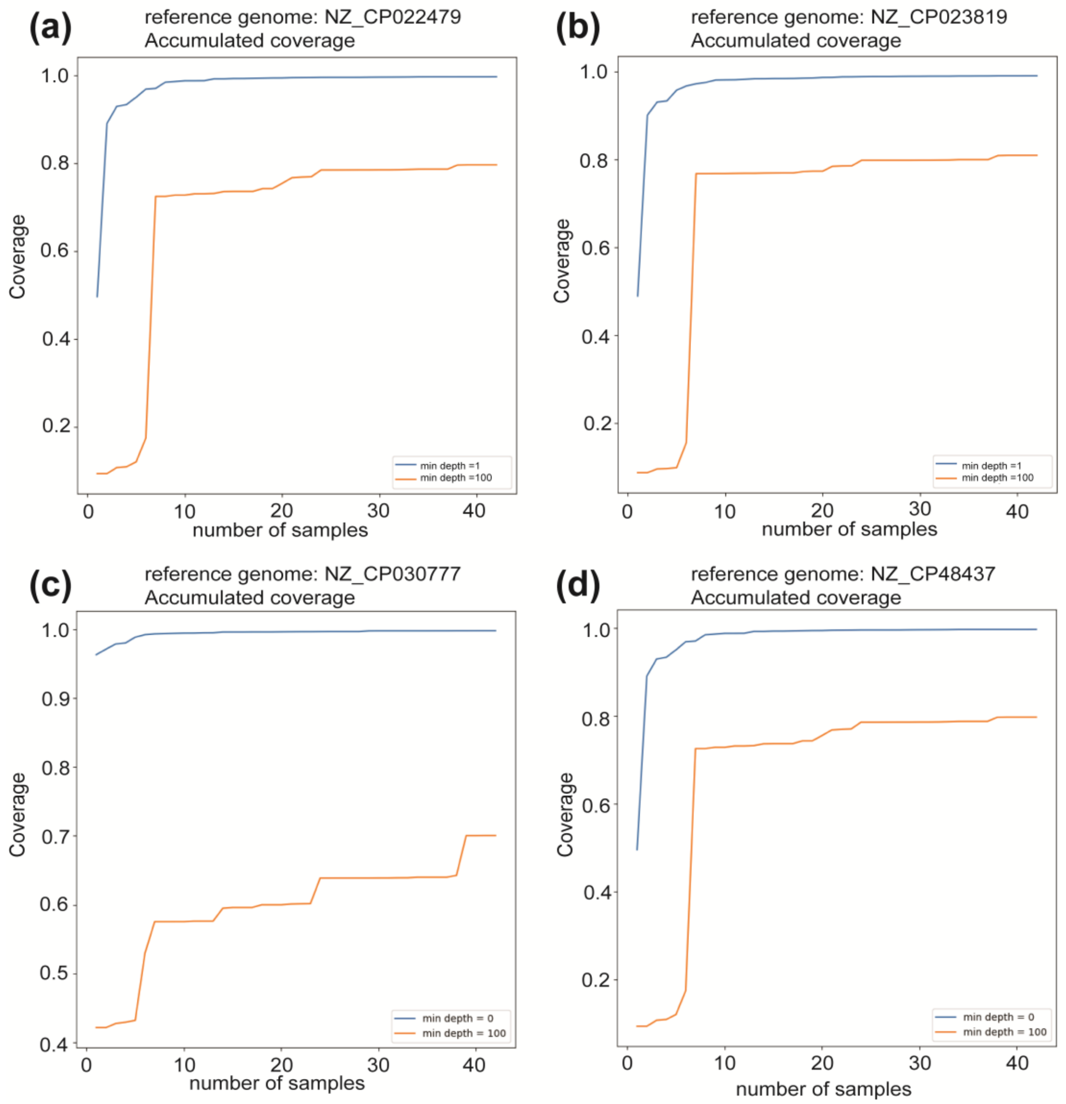

Supplementary figure 4: **Evaluating the impact of different reference genomes**. The coverage (%) of a reference genome on each sample was calculated and the relationship was visualized between the cumulative coverage and the number of metagenome samples included in a study. Both 1X (blue) and 100X (orange) minimum sequencing depth for were considered for genome coverage calculation here. (**a**) Reference genome NZ_CP022479. (**b**) Reference genome NZ_CP023819. (**c**) Reference genome NZ_CP030777. (**d**) Reference genome NZ_CP048437.

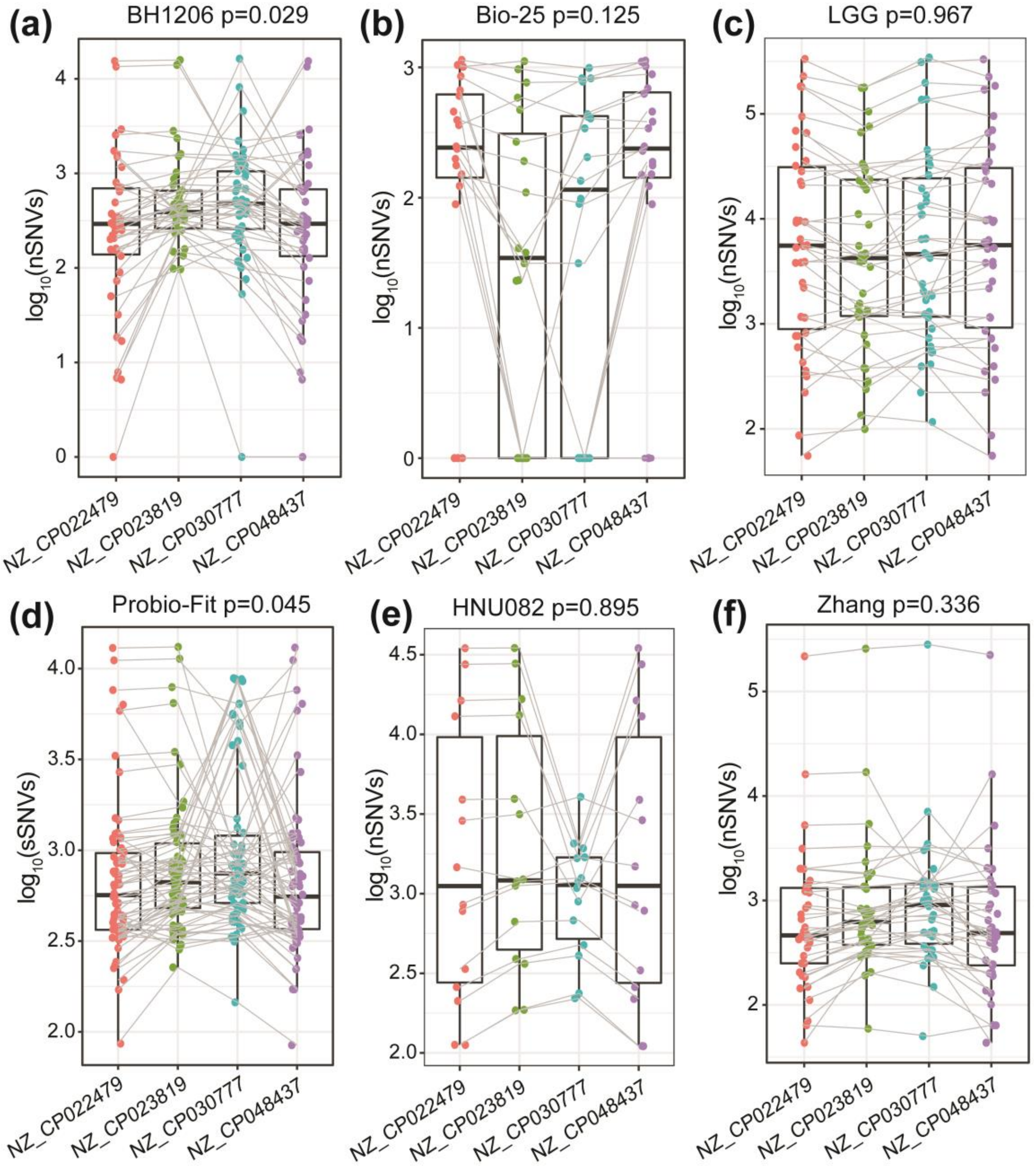

Supplementary figure 5: **Evaluating the impact of different reference genomes by SNVs numbe**r. SNVs number of four reference genomes on the six cohorts：(**a)** *B. longum* AH1206. (**b)** Supherb Bio-25. (**c**) *L. rhamnosus* GG. (**d)** Probio-Fit. (**e**) *L.plantarum* HNU082. (**f)** *L.casei* Zhang. The box represents the 25–75th percentile, whiskers represent the full range, and the line represents the median value.

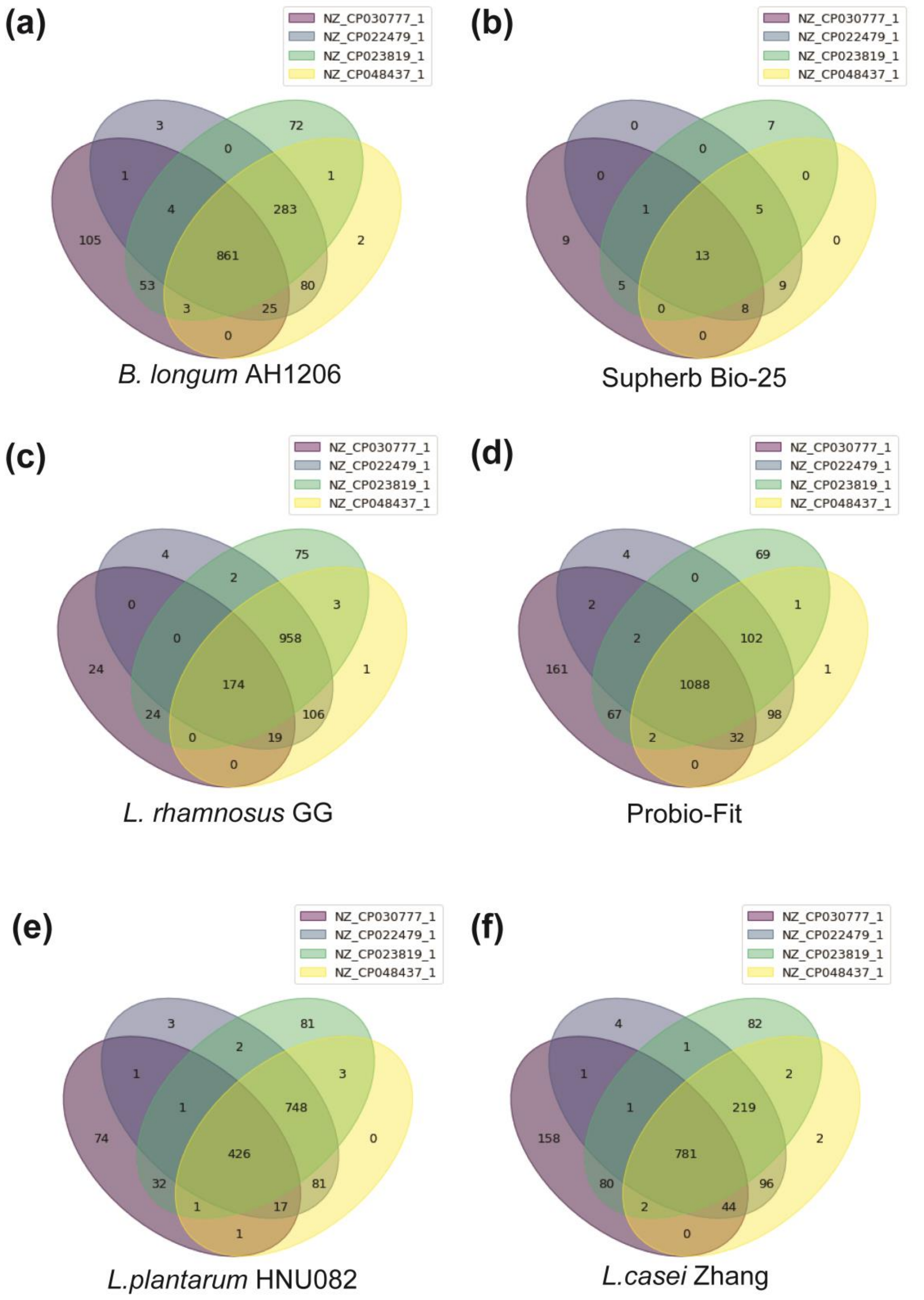

Supplementary figure 6: **Evaluating the impact of different reference genomes by gene products.** Gene products overlap of four reference genomes on the six cohorts. (**a**) *B. longum* AH1206. (**b**) Supherb Bio-25. (**c**) *L. rhamnosus* GG. (**d**) Probio-Fit. (**e**) *L.plantarum* HNU082. (**f**) *L.casei* Zhang.

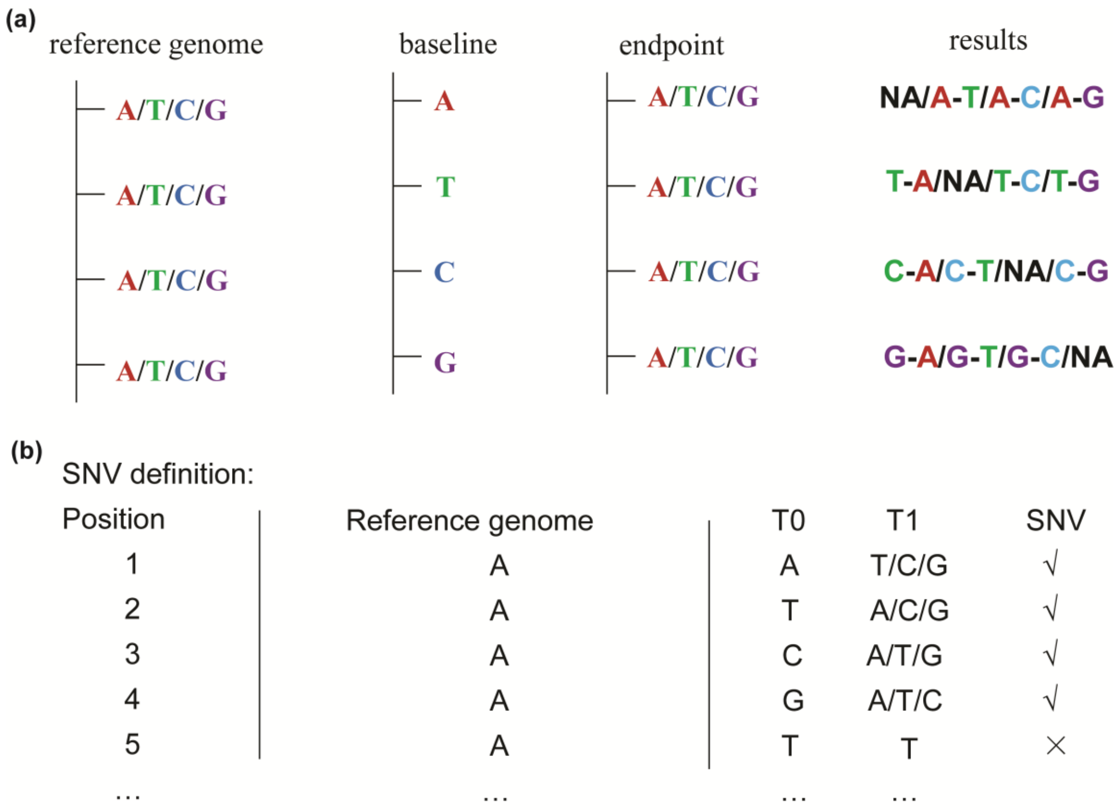

Supplementary figure 7: The details of methods about SNV definition. The reference genome is merely a bridge, and the directions of SNVs mutation were determined by the changes between the baseline and endpoint of probiotic consumptions. **(a-b)**. Any graph can understand the definition.

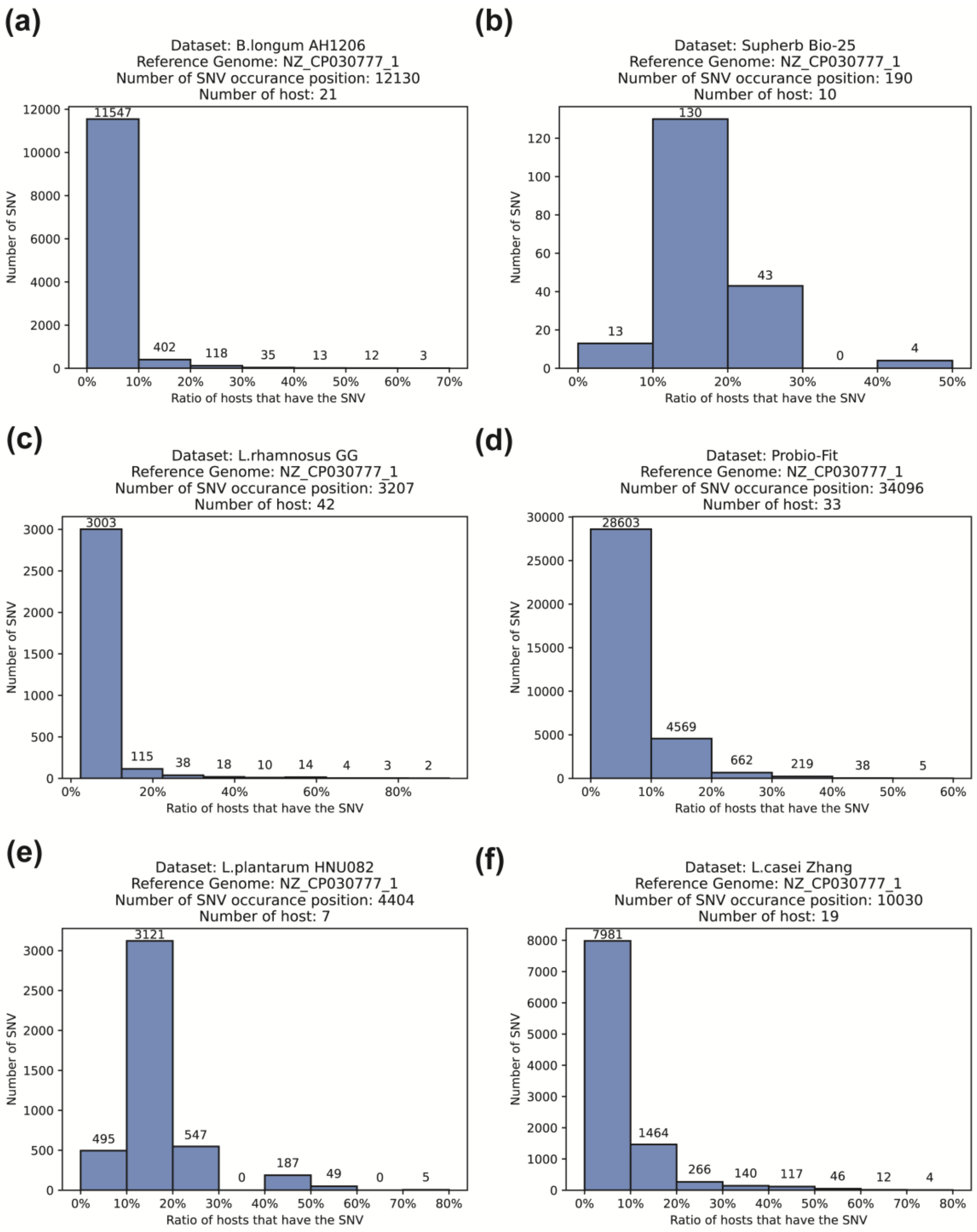

Supplementary figure 8: The proportion of each *F. prausnitzii* SNV present in the host. The figure shows the cohort, reference genome, number of SNV occurance position and number of host. The interval scale contains the right hand side. The x-coordinate is the ratio of hosts that have the SNV, and the y-coordinate is SNV number. Six cohorts were considered, including (**a)** *B. longum* AH1206. (**b)** Supherb Bio-25. (**c**) *L. rhamnosus* GG. (**d)** Probio-Fit. (**e)** *L.plantarum* HNU082. (**f)** *L.casei* Zhang.

ANI value	NZ_CP030777	NZ_CP022479	NZ_CP023819	NZ_CP048437
NZ_CP030777	100%			
NZ_CP022479	86.24%	100%		
NZ_CP023819	86.73%	97.39%	100%	
NZ_CP048437	86.38%	99.99%	97.41%	100%

Supplementary table 1. T**he Average Nucleotide Identity (ANI) values among these four genomes**. Genome-wide distance was compared between these four genomes with the Average Nucleotide Identity (ANI) values (http://enve-omics.ce.gatech.edu/ani/index).
