# Supplementary material for: Probiotic consumption influences universal adaptive mutations in indigenous human and mouse gut microbiota
Source: Commun Biol. 2021 Oct 18;4:1198. doi: 10.1038/s42003-021-02724-8 (PMC8523657; doi:10.1038/s42003-021-02724-8)
Supplement: Supplementary file 3 — Description of Supplementary files [file 42003_2021_2724_MOESM3_ESM.pdf]

Description of Supplementary Files

File name: Supplementary data 1
Description: All selected reference or representative strains from NCBI and their GenBank accessions.

File name: Supplementary data 2.
Description: All SNVs profiles in 12 cohorts due to probiotics consumption.

File name: Supplementary data 3.
Description: Quantify the effect size of environmental factors using PERMANOVA test.

File name: Supplementary data 4.
Description: The shared SNVs of the six human's probiotics intervention.

File name: Supplementary data 5.
Description: SNVs profiles of three species in Israel control cohorts.

File name: Supplementary data 6.
Description: The protein information involved in 610 HNU082-indcued SNVs.

File name: Supplementary data 7.
Description: All source data for graphs.
